# Disturbance alters relationships between soil carbon pools and aboveground vegetation attributes in an anthropogenic peatland in Patagonia

**DOI:** 10.1002/ece3.8694

**Published:** 2022-03-21

**Authors:** Javier Lopatin, Rocío Araya‐López, Mauricio Galleguillos, Jorge F. Perez‐Quezada

**Affiliations:** ^1^ Faculty of Engineering and Science University Adolfo Ibáñez Santiago Chile; ^2^ 419961 Data Observatory Foundation Santiago Chile; ^3^ Center for Climate Resilience Research (CR)^2^ University of Chile Santiago Chile; ^4^ 14655 Center for Integrative Ecology School of Life and Environmental Sciences Deakin University Melbourne Victoria Australia; ^5^ Department of Environmental Science and Renewable Natural Resources University of Chile Santiago Chile; ^6^ 14655 Institute of Ecology and Biodiversity Santiago Chile

**Keywords:** growth forms, management, plant functional types, PLS path modeling, structural equation modeling

## Abstract

Anthropogenic‐based disturbances may alter peatland soil–plant causal associations and their ability to sequester carbon. Likewise, it is unclear how the vegetation attributes are linked with different soil C decomposition‐based pools (i.e., live moss, debris, and poorly‐ to highly‐decomposed peat) under grassing and harvesting conditions. Therefore, we aimed to assess the relationships between aboveground vegetation attributes and belowground C pools in a Northern Patagonian peatland of *Sphagnum magellanicum* with disturbed and undisturbed areas. We used ordination to depict the main C pool and floristic gradients and structural equation modeling (SEM) to explore the direct and indirect relationships among these variables. In addition, we evaluated whether attributes derived from plant functional types (PFTs) are better suited to predict soil C pools than attributes derived from species gradients. We found that the floristic composition of the peatland can be classified into three categories that follow the C pool gradient. These categories correspond to (1) woody species, such as *Baccharis patagonica*, (2) water‐logged species like *Juncus procerus*, and (3) grasslands. We depicted that these classes are reliable indicators of soil C decomposition stages. However, the relationships change between management. We found a clear statistical trend showing a decrease of live moss, debris, and poorly‐decomposed C pools in the disturbed area. We also depicted that plant diversity, plant height, and PFT composition were reliable indicators of C decomposition only under undisturbed conditions, while the species‐based attributes consistently yielded better overall results predicting soil C pools than PFT‐based attributes. Our results imply that managed peatlands of Northern Patagonia with active grassing and harvesting activities, even if small‐scaled, will significantly alter their future C sequestration capacities by decreasing their live and poorly‐decomposed components. Finally, aboveground vegetation attributes cannot be used as proxies of soil C decomposition in disturbed peatlands as they no longer relate to decomposition stages.

## Graphical abstract

We aimed to assess how anthropogenic disturbances affect the relationships between aboveground vegetation attributes and belowground C pools in a peatland located in Northern Patagonia, Chile. We found that undisturbed peatland has more statistically consistent relationships between soil C pools and vegetation attributes, yielding higher predictive accuracies than disturbed areas. In addition, we observed that water‐logged plant communities have different variable interactions than dryer communities.

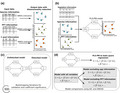

## 1 INTRODUCTION

Peatlands account for one‐third of the world's soil carbon (C) and are essential to regulate global climate (Joosten & Couwenberg, 2008). They have a high C sink capacity and store ~95% of their C pools belowground (Smith et al., 2004). The amount of C sequestered depends on the balance between gross primary production (from photosynthesis), respiration, and decomposition of plant materials. Therefore, changes in species composition may significantly affect C sequestration as species differ considerably in productivity and decomposability (Cornwell et al., 2008). Likewise, human activities, such as peat harvesting and cattle grazing, are significant factors accounting for changes in peatland floristic composition (Domínguez et al., 2012), affecting the rates of organic matter decomposition, reducing the soil carbon stock (Valdés‐Barrera et al., 2019), and releasing CH_4_ and CO_2_ into the atmosphere (Loise et al., 2020; Miettinen et al., 2017; Walker et al., 2016). Thus, accelerating the greenhouse gas effect and contributing to global warming (Phillips & Beeri, 2008; Schaepman‐Strub et al., 2008).

In contrast to the extensively studied peatlands of the Northern Hemisphere, research on Patagonian peatlands is scarce (León et al., 2018). Joosten and Clarke (2002) estimated the surface of peatlands in Chilean Patagonia to be near 10,470 km^2^, corresponding to 1.4% of the national territory (Iturraspe, 2016). On Chiloé Island, Northern Patagonia, vast expanses of forest areas were burned or cleared for timber or to prepare the land for agriculture or livestock due to accelerated human colonization. Nahuelhual et al. (2013) stated that the northern part of the island suffered a loss of 38% of the old‐growth forest area from 1976 to 2007 (equivalent to 39,013 ha). As a result, poor drainage areas were colonized by *Sphagnum* mosses (mainly *S. magellanicum*) or successional shrublands (Díaz et al., 2007). These peatlands are called “anthropogenic peatlands” (Díaz et al., 2008; León et al., 2018).

Given the recent nature of their formation, anthropogenic peatlands differ substantially from natural peatlands in flora and levels of carbon storage (Díaz et al., 2008). For instance, the average C accumulation rate in anthropogenic peatlands of Northern Patagonia is higher (107.3 g C m^−2^ yr^−1^) than natural Patagonian peatland (78.3 g C m^−2^ yr^−1^) (León et al., 2018). Moreover, the C accumulation rate in anthropogenic peatland was significantly higher in young peat deposits near the surface (507.52 g C m^−2^ yr^−1^) in comparison with natural peatland (335.58 g C m^−2^ yr^−1^) (León et al., 2018). These differences may be attributed to the unsaturated conditions of young peat deposits near the surface, leading to a greater rate of C accumulation than old peat, and potentially due to the differences in their floristic composition. Anthropogenic peatlands of Northern Patagonia are commonly harvested for the superficial layer of *S. magellanicum* moss for horticultural purposes and used for cattle grazing (Cabezas et al., 2015; Díaz et al., 2008). These activities have been shown to increase the species richness, facilitate the arrival of exotic species, and reduce their soil carbon sink capacity (Cabezas et al., 2015; Domínguez et al., 2012; Valdés‐Barrera et al., 2019).

Anthropogenic perturbations on peatlands often have a cumulative effect: when combined, the impacts are more significant than those expected from each disturbance operating separately (Limpens et al., 2008). Nevertheless, it is still unclear how these disturbances further alter the interactions between belowground C pools, including live mosses, debris, and different peat layers, and aboveground vegetation attributes, like vegetation height, biomass, and community composition (Hapsari et al., 2018; Yang et al., 2017). Likewise, dominant vegetation with different plant functional types (PFTs) or growth forms provides a wide variety of food sources with varying quality for belowground decomposers, affecting carbon turnover (e.g., Chen et al., 2016). For example, peatland mosses, shrubs, and sedges produce a litter with different lignin and polyphenol contents, resulting in varying effects on soil respiration and organic matter decomposition rates (Ward et al., 2009). Likewise, Dorrepaal (2007) found that differences in leaf litter decomposability are large between broad PFT groups (vascular and non‐vascular plants) but small and environment‐dependent among narrow vascular growth forms (*Sphagnum*, evergreen‐shrubs, deciduous‐shrubs, forbs, and graminoids). However, it is still unclear if these PFTs are reliable indicators of decomposition stages in anthropogenic peatlands under grazing and harvesting. Hence, accounting for C stored in peatland and its decomposition stages are essential for conservation and monitoring purposes.

This study aims to assess the effects of anthropogenic pressure on peatland structure and functions and its associated C dynamics. We hypothesized that sustained grazing and moss harvesting leads to differences in the relationships between aboveground vegetation attributes and belowground C pools. The study site corresponds to an anthropogenic peatland in Northern Patagonia with two different managements: disturbed and undisturbed. We further asked the following questions:

1. Which components of the soil C pool continuum differ significantly between the undisturbed and disturbed peatland?
2. How do the main floristic gradients relate to soil C decomposition?
3. Which relationships between aboveground vegetation attributes and belowground C pools differ significantly between the undisturbed and disturbed peatland?
4. Do PFT‐based outperform species‐based attributes to predict soil C pools in the study area?

## 2 MATERIALS AND METHODS

### 2.1 Study area

**FIGURE 1:**
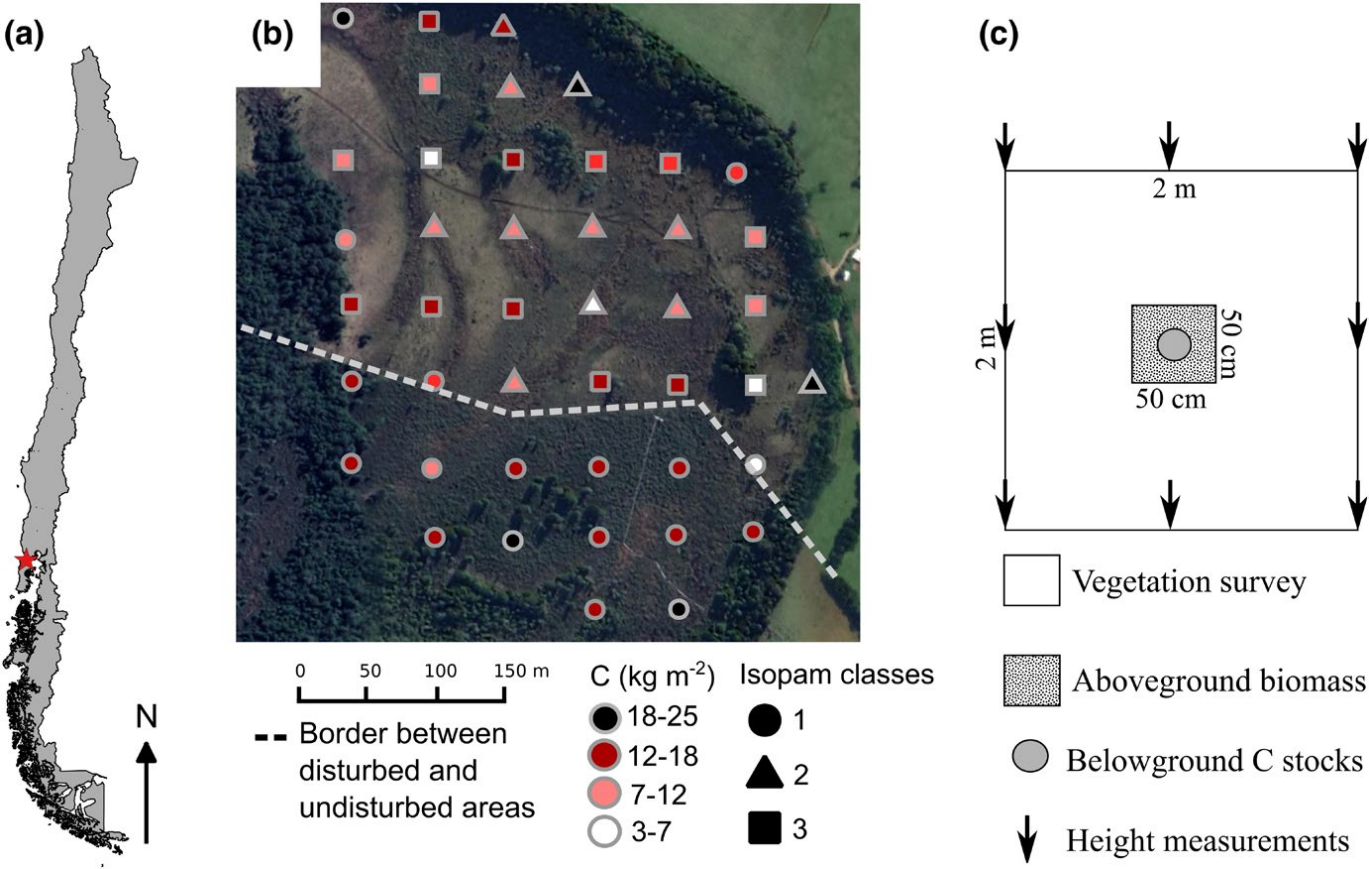
(a) Location of Senda Darwin peatland; (b) High‐resolution RGB composite image of the study area with plot colors scaled with the measured belowground C stocks (sum of C pools) and the Isopam classes. The dashed line shows the border between the disturbed area (upper) and undisturbed (bottom) areas; (c) detailed sampling design

The study site is an anthropogenic peatland located at the FLUXNET site *Senda Darwin Peatland* (CL‐SDP; 41°52′S, 73°40′W), north of Chiloé Island, Los Lagos region of Chile (Figure 1a). North patagonia presents many similar isolated peatlands, which, despite their relatively low individual area, sum up to considerable amounts of total C stocks (León et al., 2018).

The peatland originated from a forest fire that occurred >50 years ago (Perez‐Quezada et al., 2021). The peatland is about 16 ha, and it is divided into two areas (Figure 1b): an undisturbed area (~5.5 ha) that has been protected and used for scientific investigations during the last 26 years and a disturbed area (~10.5 ha) that has been used used for artisanal extraction of *Sphagnum* mosses for commercial purposes (~10 kg of dry moss per month during >50 years) and grazing of four oxen. Both grazing and artisanal extractions are common practices in the anthropogenic peatlands of Chiloé, which cover about 8% of the landscape. Nowadays, the undisturbed area behaves like a small C sink, while the disturbed area is very close to C neutrality but could turn into a C source if degradation continues (Valdés‐Barrera et al., 2019).⁠

The floristic composition follows a gradient of water availability (León, 2012). We assessed homogeneous floristic composition classes using the Isopam clustering algorithm (i.e., classification of ordination scores from isometric feature mapping; Schmidtlein et al., 2010) and found three main floristic categories in the peatland (Figure 1b). The first occurs mainly in the undisturbed area, where the dominant species are *Baccharis patagonica*, *Sticherus cryptocarpus*, *Myrteola nummularia*, and *Blechnum penna*‐*marina* ferns. The second class corresponds to permanently waterlogged areas; common species are *Juncus stipulatus*, *Juncus procerus*, and *Carex magellanica*. Finally, the third class is primarily present in the disturbed area, where the dominant species are *Carex distenta*, *Danthonia chilensis*, and exotic species (e.g., *Leontodon saxatilis*). Generally, classes one and three are located in higher hummocks, while class two (water‐logged species) are located in low hummocks. *Sphagnum magellanicum* is present in the three classes but more abundant in the first class (complete list of species in Appendix S1 of Cabezas et al., 2015).

### 2.2 Field data

We used the data collected by Cabezas et al. (2015), including a vegetation assessment (between January and April 2014) in 44 plots placed in a 60 m × 60 m systematic grid spacing, where 19 of the plots were located in the disturbed area and 15 in the undisturbed area. At each point, we established a 2 m × 2 m plot to estimate species presence, cover, biomass, vegetation height, and the soil C pools. Species cover was obtained by averaging visual estimates of two observers. Aboveground biomass was estimated by harvesting the vascular flora in a 0.25 m^2^ sub‐plot located in the plot's center (Figure 1c).

The underground C pools were extracted using cylindrical samples (52 mm diameter) at the plot's central point using a peat profile sampler (Eijkelkamp, Giesbeek, Nether‐lands). We measured until the end of each plot's soil profile, so the general sampling soil depth was variable. We categorized the decomposition stages using the methodology proposed by Von Post (1924). Each peat core was subdivided into three classes (i.e., R1, R2, and R3; Table 1) (Cabezas et al., 2015; Monsalve et al., 2021; Savvas & Passam, 2002). We further kept the live moss and fine and coarse debris as separate pool classes (Cabezas et al., 2015). See Table 1 for reviewing the belowground pools. Then, we estimated the soil layer densities of the live moss, fine and coarse debris, R1, R2, and R3 C pools from samples of known volume. We then dried all samples at 70°C for 72 h and weighed all C pools separately. Finally, we depicted five random sub‐samples of 10 g from each C pool (Cabezas et al., 2015). These sub‐samples were ground and later analyzed with an elemental analyzer to characterize live moss, fine and coarse debris, R1, R2 C and biomass stocks independently (NA2500, Carlo Erba, Milan, Italy).

**TABLE 1:** Belowground carbon pools and their characteristics

Pool	Plant parts	Characteristics of water squeezed from peat sample	Von post	Peat characteristics	C pools (kg m^−2^)	
					Undisturbed area	Disturbed area
Fine debris	Trunks or branches <5 mm in diameter	–	–	–	0.27 ± 0.1	0.03 ± 0.03
Coarse debris	Trunks or branches >5 mm in diameter	–	–	–	0.13 ± 0.1	0.00 ± 0.00
Live moss	Plant individuals fully recognizable	Colorless, almost transparent, and slightly cloudy	–	–	0.14 ± 0.06	0.09 ± 0.04
R1	From completely undecomposed peat to very slightly decomposed peat	From almost clear water to muddy brown water	H1–H3	Plant remains identifiable, and no amorphous material present	0.26 ± 0.15	0.49 ± 0.22
R2	From slightly decomposed peat to moderately‐highly decomposed peat	Maximum one‐third of the peat escapes between the fingers	H4–H6	The residue is very pasty but shows the plant structure more distinctly than before squeezing	0.86 ± 0.41	0.97 ± 0.36
R3	From highly decomposed peat to completely decomposed peat	More than one‐half of the peat escapes between the fingers	H7–H10	Very faintly to no discernible plant structure	10.39 ± 1.66	9.55 ± 1.17

### 2.3 Modeling approach

**FIGURE 2:**
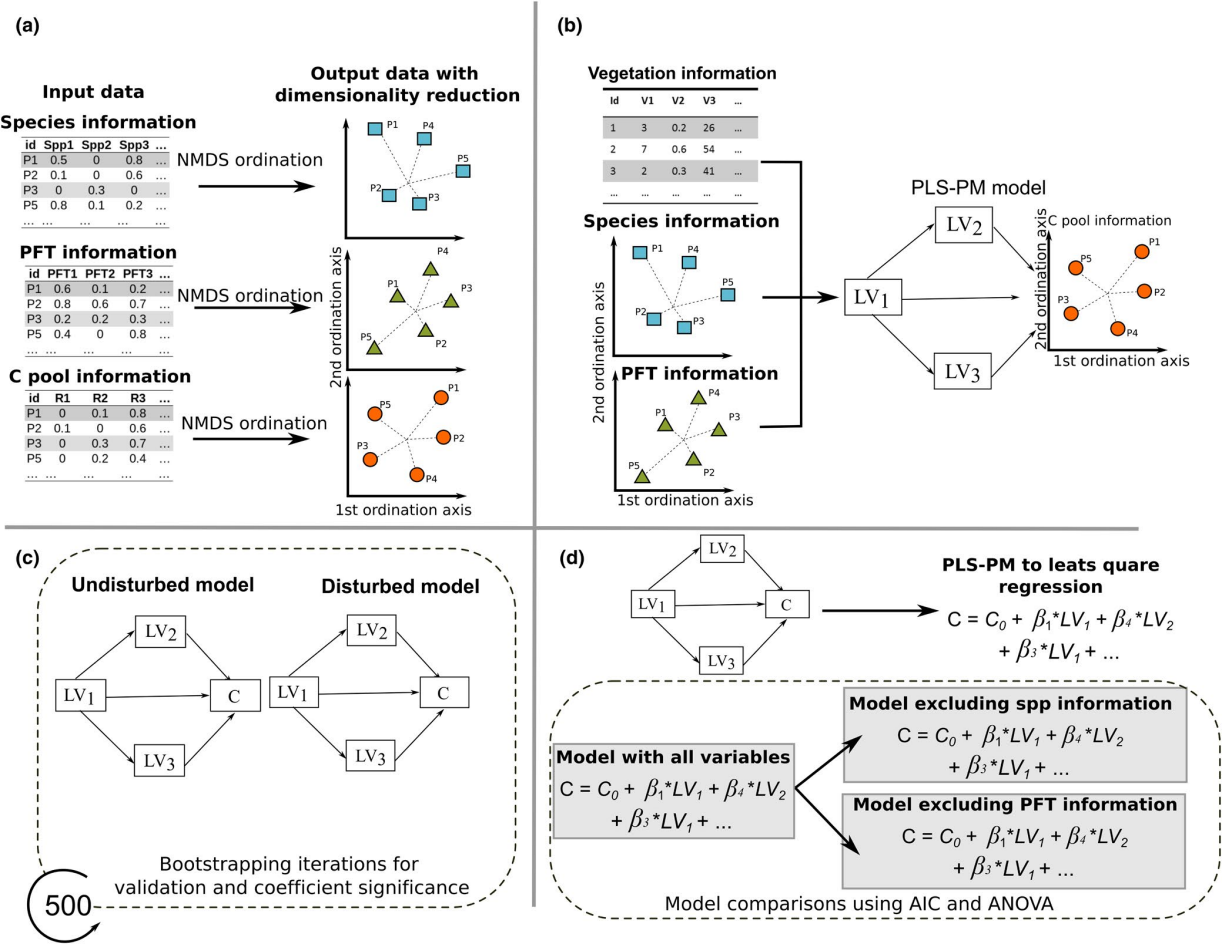
Proposed modeling approach. (a) Dimensionality reduction of species, plant functional types (PFT), and C pools using Nonmetric Multidimensional Scaling (NMDS) ordinations. (b) Definition of a structural equation model (SEM) for disentangling the relations between vegetation attributes and soil C pools using plot‐based measurements and the ordination‐based components (a). (c) Comparison of causal links of the SEM between disturbed and undisturbed areas of the peatland. (d) Comparison of predictive accuracy between a regression model using all vegetation variables and models excluding species‐based and PFT‐based information

The proposed modeling approach can be summarized into four main steps (Figure 2):

1. Definition of vegetation predictors. Here we applied ordination transformations to floristic, plant functional types (PFT), and C pool information for dimensionality reduction (Figure 2a).
2. Definition of a structural equation model (SEM). Here we defined an a priori structural model based on literature review and aimed for disentangling the effects of aboveground vegetation attributes in belowground C pools. Data input comprises plot‐based vegetation attributes and C pool information with dimensionality reduction (output of step 1; Figure 2b).
3. Characterization of causal links between vegetation attributes and belowground C pools in the disturbed and undisturbed areas of the peatland. Here we used the SEM defined in step 2 (Figure 2c).
4. Characterization of the relative importance of species‐ and PFT‐based community composition. Here we fitted regression models using all vegetation variables and compared their accuracies with models excluding species‐based and PFT‐based information (Figure 2d).

#### 2.3.1 Model variables and ordination transformations

We selected five types of aboveground vegetation attributes linked with belowground C pools: (i) vegetation height, (ii) plant diversity, (iii) aboveground biomass, (iv) the assemblages of vascular species communities, and (v) the assemblages of plant functional types (PFTs). These variables are directly or indirectly related to the decrease of water‐logging conditions and peat mineralization in disturbed and undisturbed peatlands, causing the decline of *Sphagnum* cover and promoting colonization by vascular plants. We considered the PFTs as the bryophytes, graminoids, forbs, and shrubs’ growth forms, as this classification showed strong relationships with carbon cycling feedbacks (Dorrepaal, 2007). We estimated the Shannon‐Wiener index using the species and PFT abundance matrices for the plant diversity component. Aboveground biomass components consisted of herbaceous (i.e., forbs + graminoids) and shrub components (kg m^−2^).

We used ordination transformations to depict the main floristic, PFT, and C pool compositions using the Nonmetric Multidimensional Scaling (NMDS; Shepard, 1962; Figure 2a). NMDS reduced data multidimensionality by creating a fixed number of components summarizing the main data gradients. The final number of components for the species, PFTs, and C pool information were selected by keeping the model *‘stress’* values below 1.5 (using the best solution of 500 iterations; Paliy & Shankar, 2016). The species‐ and PFT‐based ordinations used the plot‐by‐species and ‐PFT matrices as input to depict continuous gradients of plant community transitions. Meanwhile, the NMDS‐based dimensionality reductions of belowground C pools data used the six measured C pools as input (Table 1), that is, live moss, fine and coarse debris, R1, R2, and R3, to obtain continuous gradients of C decompositions. We used the Bray‐Curtis dissimilarity distance in all cases as it is robust while dealing with datasets with a significant presence of zeros (Shepard, 1962), which was the case of these three data types.

Finally, we used one‐sided ANOVA tests to check for significant differences in the C pool components between the undisturbed and the disturbed area. We used the R‐package “*vegan*” (Oksanen et al., 2016) (scripts available, see *Data availability statement*).

#### 2.3.2 Modeling variable causal effects via structural equation modeling

We used structural equation modeling (SEM) to link the created C pool components to the aboveground vegetation attributes, creating a theoretical model of direct and indirect cause‐effect relationships (Figure 2b). We applied partial least squares path modeling (PLS‐PM; Tenenhaus et al., 2005), a non‐parametric composite‐based SEM that has shown robustness while dealing with complex ecological data (Ferner et al., 2018; Lopatin et al., 2015, 2019; Perez‐Quezada et al., 2021). We standardized the latent variables (LVs) to normalize path coefficients and intercepts (Grace & Bollen, 2005) and tuned the algorithm by using the Cronbach's alpha index (check for unidimensionality among indicators) and the loading values (correlation within the indicators of an LV; we dropped variables with loadings below 0.5). We used bootstrapping with 500 iterations as independent validation for model assessment (*R* ^2^ and goodness‐of‐fit) and to depict significant path coefficients (α = 0.05).

We used multiple ordinary least square regressions to select the species‐ and PFT‐based components included during modeling (e.g., Grace et al., 2016). We trained and compared two models (Figure 2c):

1. Models using only data at the undisturbed area
2. Models using only data at the disturbed area

We repeated this analysis for the selected NMDS‐based C pool components (Figure 2a). We used Moran's *I* index to check for spatial autocorrelation on the residuals of the LVs (Lopatin et al., 2019; Perez‐Quezada et al., 2021). Finally, we used the R‐package “*plspm*” (Sanchez et al., 2017) for the analysis (scripts available, see *Data availability statement*).

#### 2.3.3 Differences between species‐ and PFT‐based information

Finally, we tested if the effects of the species‐based community composition on the models statistically differ from the effects of the PFT‐based gradients. For this test, we compared linear regressions models predicting the C pool components using all PLS‐PM‐based vegetation components with models first excluding the species‐based compositions and then the PFT‐based gradients. We used the Akaike Information Criterion (AIC) to depict the best models and a one‐sided ANOVA test to assess the significant levels. We tested normality in the model's residuals using the Shapiro–Wilk normality test (Royston, 1982).

## 3 RESULTS

### 3.1 Which components of the soil C pool continuum differ significantly between the undisturbed and disturbed peatland?

**FIGURE 3:**
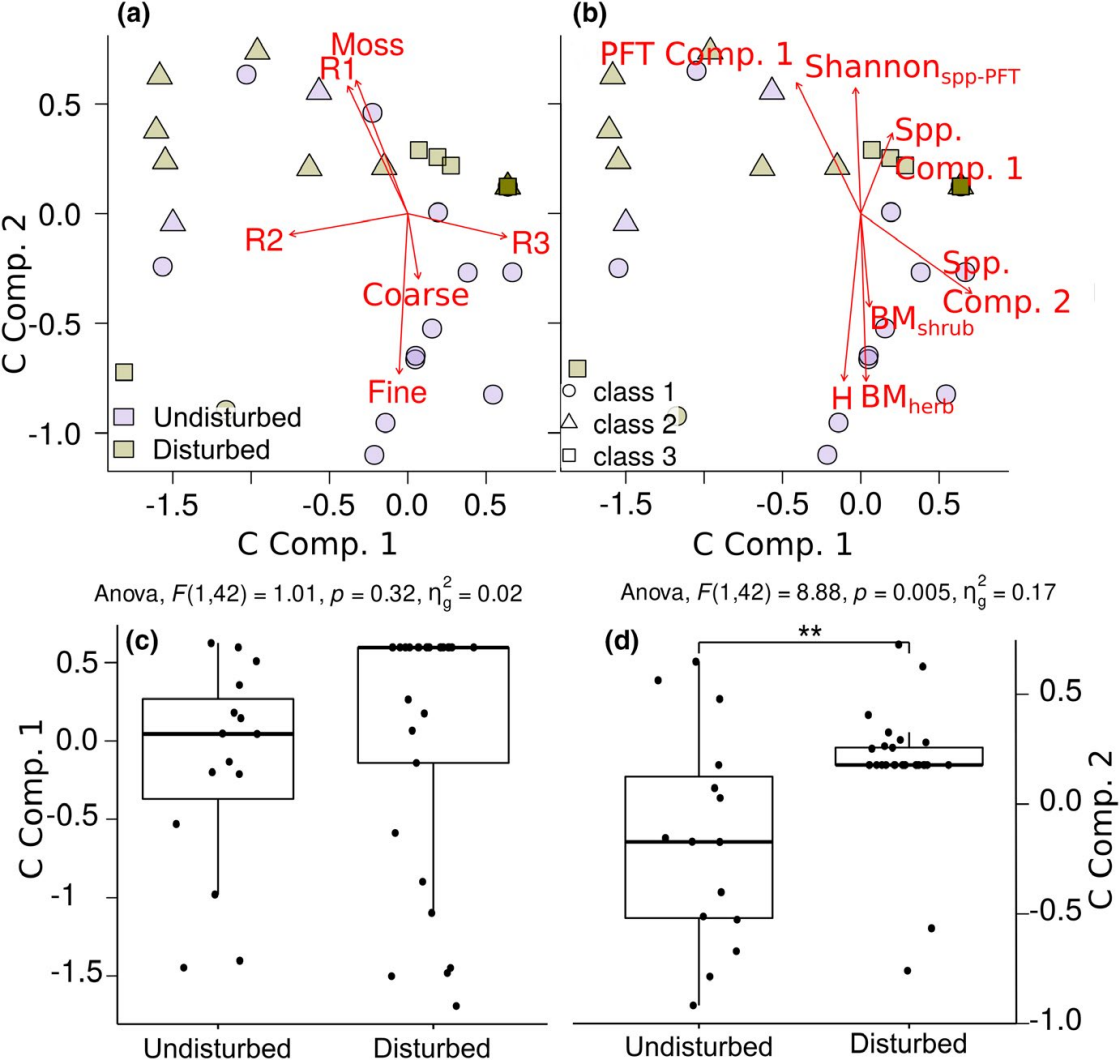
(a, b) Components of belowground C pools using Nonmetric Multidimensional Scaling (MNDS). Close plots feature more similar species compositions than remote plots. Vectors illustrate the correlations of the NMDS axes with the variables (Table S1). (c, d) Distribution of values of the C Comp. 1 and C Com. 2 in the disturbed and undisturbed areas, and results from the ANOVA test

The distribution of the six belowground C pools in the feature space reflects two main gradients, which result in similar eigenvalues and axis limits (Figure 3a‐b). However, the second axis (C Comp. 2) separates classes and perturbation site conditions. The first C axis (C Comp. 1) shows moderate to complete decomposition C components (R2→R3). Meanwhile, C Comp. 2 shows undecomposed to slightly decomposed C components, from fine‐coarse debris (low axis values) to R1 and live mosses (high axis values). In general, less decomposed soil C pools are associated with the undisturbed site (fine and coarse debris), while live moss and slightly decomposed soil C pool (R1) with the disturbed site (C Comp. 2). However, this difference is less clear for the C comp.1, accounting for moderately and highly decomposed soil C components. We found the only clear statistical differences between the disturbed and undisturbed peatland in the C Comp. 2 (Figure 3d).

### 3.2 How do the main floristic gradients relate to soil C decomposition?

**FIGURE 4:**
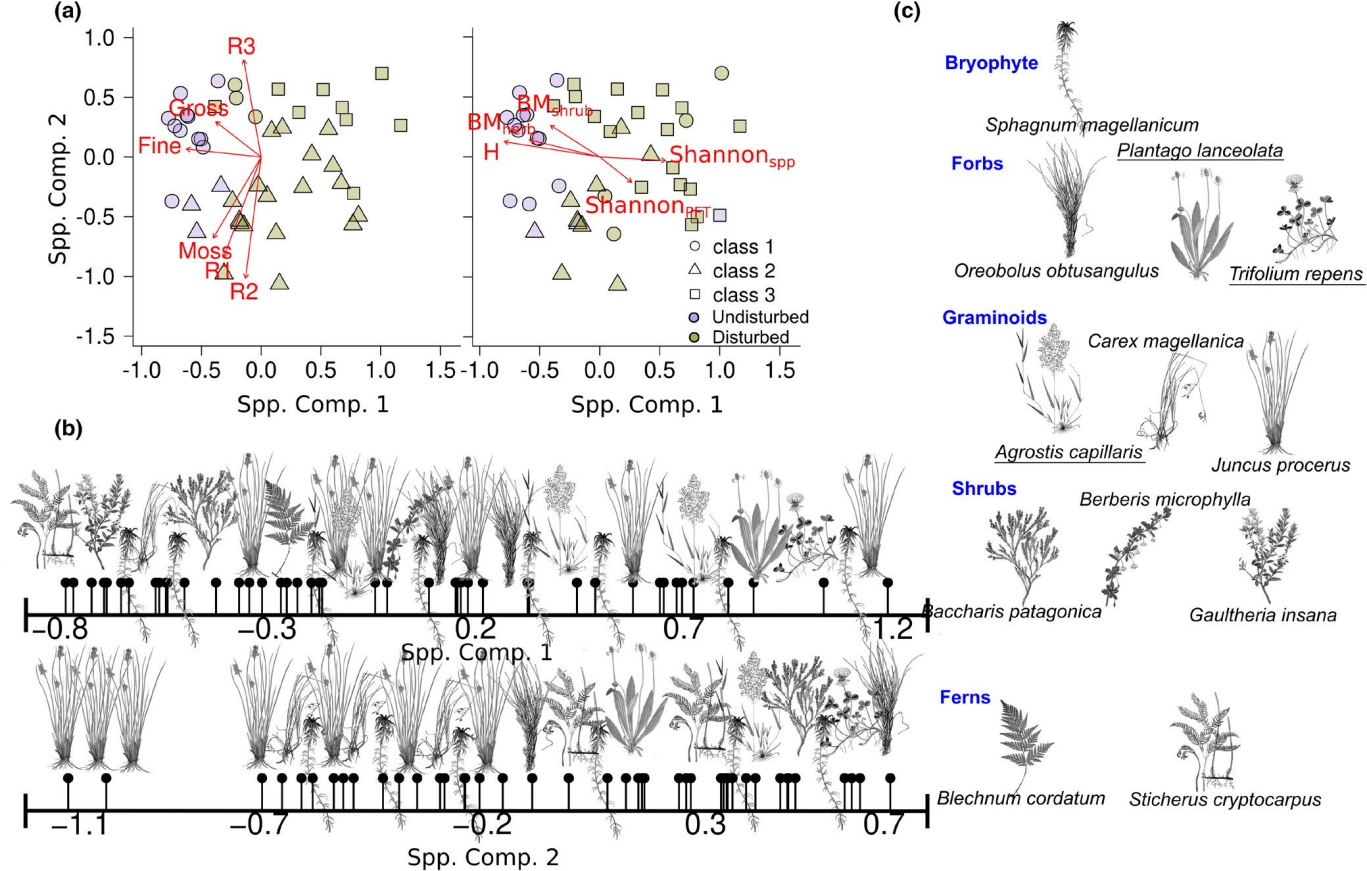
Peatland Species‐based floristic gradients. (a) Distribution of plots (sample points) in the two‐dimensional ordination space of Nonmetric Multidimensional Scaling (NMDS), showing the gradients of the belowground C pools (left) and the management types and Isopam classes (right). Close plots feature more similar species compositions than remote plots. Vectors illustrate the correlations of the NMDS axes with the variables (Table S1). (b) Location of the most typical species and field‐plots (black dots) along the first two NMDS axes. (c) Dominant species were identified with the Isopam clustering algorithms. Underlined species names indicate exotic species

Figure 4 presents the species‐based floristic composition of the 49 peatland species, and Table S1 summarizes all derived vegetation attributes. We selected two components after the multiple regression analysis (Table S2 in Appendix S1). The most common species are shown in Figure 4b,c. The first NMDS species‐based axis (Spp. Comp. 1) depicted the most significant floristic gradient, with community assemblages of the undisturbed peatland at the low‐end and the disturbed area at the high‐end (threshold at ~−0.5). This gradient shows significant variability of fine and gross soil C debris, aboveground biomass, vegetation heights towards the undisturbed area (negative axis values; Isopam class 1), and species‐ and PFT‐based diversity to the disturbed area (positive axis values; Isopam classes 2 and 3).

The second NMDS species‐based axis (Spp. Comp. 2) is associated with water‐logged plant communities (Isopam class 2) at the low‐end and dryer plant communities (Isopam classes 1 and 3) at the high‐end. This water‐logged communities indicates poorly‐ and moderate‐decomposition stages, like live mosses, R1, and R2. Likewise, we found that plant diversity is related to live moss and R1 components (i.e., less decomposed). In contrast, vegetation heights and biomass are related to fine and coarse debris. Meanwhile, R2‐R3 gradients relate to water‐logged to dryer plant community transitions (second species‐based floristic component).

Regarding the PFT‐based gradient (Figure S1 in Appendix S1), we found that the first component axis relates to vegetation variables similar to the species‐based gradient: aboveground biomass and vegetation height on the negative axis values and diversity on the positive axis values. It also depicts the high soil C decomposition components and debris on the negative values and low to moderate C components on the positive values.

### 3.3 Which relationships between aboveground vegetation attributes and belowground C pools differ significantly between the undisturbed and disturbed peatland?

**FIGURE 5:**
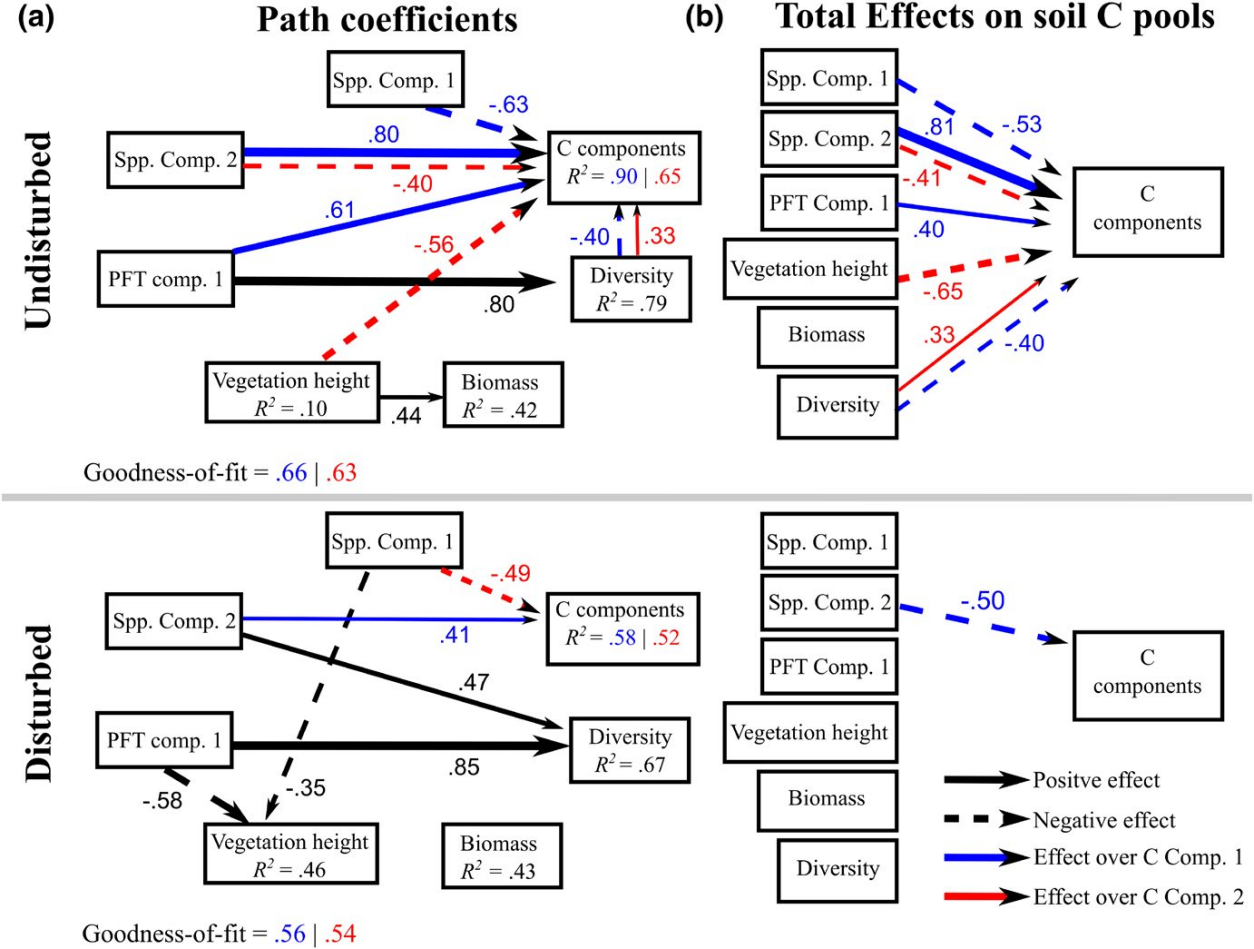
PLS‐PM results for the undisturbed and disturbed models. Arrows represent significant path coefficients among LVs, with solid and dashed arrows denoting positive and negative relationships, respectively. Black arrows show repeated model direct effects, while blue and red for C Comp. 1 and C Comp. 2, respectively. Path coefficient thickness is scaled based on its magnitude. Only significant relationships are presented (α = 0.05)

The PLS‐PM models yielded accurate overall goodness‐of‐fit, ranging from 0.54 (C Comp. 2 in the disturbed area) to 0.66 (C Comp. 1 in the undisturbed model). Meanwhile, the explained soil C pool variance (*R* ^2^) ranges between 0.52 (second component in the disturbed model) and 0.90 (first component in the undisturbed area) (Figure 5a). The undisturbed models depicted more direct significant effects on the C pool components than the disturbed models (Figure 5a). Although the relationships between vegetation attributes and C components varied in the undisturbed models, only the biomass did not show a clear statistically significant relationship with the C pool components (see the overall model in Figure S2). In contrast, the disturbed area depicted only clear significant relations with the floristic composition. The PLS‐PM model for the overall data (disturbed and undisturbed together) is presented in Figure S2 (Appendix S1). Finally, we observed spatial autocorrelation problems in the residuals only in the predictions of the C Comp. 1 of the disturbed area (see Table S3 for the detailed Morans’ *I* and *p*‐values).

The model implies moderately‐ to highly‐decomposed (R2→R3; C Comp. 1) C are explained by the increase in dryer plant communities ([+]Spp. Comp. 2) in both the disturbed and undisturbed peatland. However, the undisturbed peatland depicted significant relationships between R2‐R3 C decompositions and a mix of shrub ([‐]Spp. Comp. 1) and forb communities ([+]PFT Comp. 1) with few plant diversity ([‐]Diversity→C Comp. 1). Meanwhile, fresh and poorly‐decomposed C pools relate more to water‐logged plant communities ([‐] Spp. Comp. 1), less vegetation height, and higher plant diversity ([+]Diversity) in the undisturbed peatland, and with woody species ([‐]Spp. Comp. 1) in the disturbed area.

When considering the direct and indirect variable relationships in the models (Figure 5b), the clear statistical relationships between the vegetation proxies and belowground C pools are similar to the direct effects. However, in this case, the C Comp. 2 of disturbed models did not depict any significant link with the vegetation attributes.

### 3.4 Do PFT‐based outperform species‐based attributes to predict soil C pools in the study area?

Finally, Table  2 shows the pairwise tests between the effects of the species‐ and the PFT‐based community compositions on the PLS‐PM belowground C pools predictions. Species‐based ordination systematically explained more belowground C pools variance than PFT‐based ordination (i.e., lower AIC values). However, these differences were only significant (α < 0.001) in the undisturbed models for the moderate to completely decomposed C components (R2→R3; C Comp. 1), and in the disturbed model for fresh and poorly‐decomposed C pools (Moss→R1; C Comp. 2; *alpha* = 0.01).

**TABLE 2:** Pairwise tests of differences between the effects of the species‐ and the PFT‐based community compositions on the PLS‐PM belowground C pools predictions

	All variables	Without PFT Comp.		Without Spp. Comp.	
	AIC	AIC	*p*‐Value	AIC	*p*‐Value
Differences in the C Comp. 1					
Undisturbed model	24.30	30.63	.04*	50.14	<.001***
Disturbed model	78.39	77.78	.31	79.12	.16
Differences in the C Comp. 2					
Undisturbed model	42.81	41.47	.57	42.16	.41
Disturbed model	85.77	83.90	.75	89.25	.**05**

Note

Significant codes: ***0.001; **0.01; *0.05; 0.1 are shown in bold numbers.

## 4 DISCUSSION

Our results emphasize the apparent management‐based differences in the linkages between soil C pools and vegetation attributes and the importance of plant community composition in their causal associations. The floristic composition is strongly affected by management and the site's microtopography, including water‐logged (hollows) and dryer‐high areas (Mathijssen et al., 2019). Similarly, our study site showed significant differences in floristic compositions and plant diversity between the undisturbed and disturbed areas. The undisturbed area consisted of species‐poor communities with vascular shrubs dominated by *Baccharis patagonica*, *Sticherus cryptocarpus*, and *Myrteola nummularia*. These are the most common peatland species in the Chiloé island (Díaz et al., 2008) and often indicate an advanced vascular successional state (Castillo‐Riffart et al., 2017; Díaz & Armesto, 2007). However, these dense shrub covers could restrain the peatlands’ ecological succession as shrubs intercept less precipitation than trees, causing flooding conditions that prevent tree species from colonizing the peatland (Díaz & Armesto, 2007; Díaz et al., 2008). These naturalized ecosystems are small C sinks, sequestering roughly ~ −135 ± 267 g CO_2_ m^−1^ year^−1^ (Valdés‐Barrera et al., 2019). In contrast, the disturbed area has species‐rich communities, with dominant forb and graminoid species like *Carex distenta*, *Leontodon saxatilis*, and *Danthonia chilensis*. Here, continuous overgrazing has degraded the soil, causing the invasion of forbs and graminoids, including exotic species like *Trifolium repens*, *Plantago lanceolata*, and *Agrostis capillaris*. Valdés‐Barrera et al. (2019)⁠ found that these degraded peatlands still act as small C sinks but are close to C neutrality (~ −33 ± 111 g CO_2_ m^−1^ year^−1^). However, it is unclear how long these ecosystems will take to turn into C sources.

Previous studies agreed that plant community variability led to differences in long‐term peat and carbon accumulation (e.g., Loisel & Yu, 2013; Mathijssen et al., 2019; Ward et al., 2015). Our findings indicate that species community composition directly and indirectly influences belowground C pools during humification (Figure 5). In general, the soil C pools were larger in dryer shrub‐dominant communities than in *Juncus*’ water‐logged communities. As water‐logged plant communities often have anaerobic conditions that inhibit C decomposition (Beer et al., 2008), we found that *Juncus* areas have high live (moss) and intermediate decomposition C components. However, many *Juncus* areas, especially in the disturbed peatland, did not show highly‐decomposed peat, indicating either a low decomposition rate or due to flooding, which encourages dissolved C transport (Mulholland, 2003).

The growth form (PFT) community component was only significant for intermediate to high C decompositions in the undisturbed area. Contrary to previous research (Dorrepaal et al., 2005; e.g., Ward et al., 2009), the pairwise test of significance showed consistently better results for the models considering the species‐based community composition information over the PFT‐based information (Table 3). However, we found clear statistical differences between the species‐ and PFT‐based composition to predict R2‐R3 stages in undisturbed peatlands and debris allocation in disturbed peatlands (Table 3). Likewise, the SEM model did not depict clear statistical interactions between the PFT‐based community composition and the soil C pool allocation in the disturbed model (Figure 5). This shows that PFT‐based generalization may not describe C decompositions in areas with intense anthropogenic degradation. However, different PFT classifications may provide better linkages with soil C pools, such as groups describing more detailed water‐logged gradients (Figure 5 and Juan‐Ovejero et al., 2020).

We found that vascular plant diversity was negatively associated with peatland's fine and coarse debris and positively with poorly‐decomposed C components in the undisturbed area (Figure 5a). The negative influence of vascular plant diversity in soil C reservoirs has been associated with increased soil oxygenation, microbial activity, and the organic matter decomposition rate. Vascular plant litter is less recalcitrant than *Sphagnum* litter and decomposes faster and showed higher productivity over peat mosses (Holl et al., 2019; Scheffer et al., 2001; Thormann et al., 1999). These interactions eventually facilitate the release of ancient carbon as CO_2_ into the atmosphere (Walker et al., 2016). This process may be enhanced under current climate change scenarios, as increasing temperatures promote drought and accelerate colonization by vascular plants, increasing the microbial activity (Fenner & Freeman, 2011). However, aboveground biomass was only significant for moderate to highly‐decomposed soil C in the overall model (Figure S2). Hence, a larger biomass gradient could be needed to assess the importance of biomass in C decomposition accurately (Lopatin et al., 2019).

It is worth noticing that the statistical linkages between aboveground vegetation attributes and soil pools may be due to a legacy of past vegetation types. For example, after the burn of the original temperate rainforest, succession, and colonization of *Sphagnum* species took place (Díaz et al., 2007). Hence, the current state of the soil C pools could reflect, at least to some degree, this successional process instead of the actual community characteristics. Unfortunately, we did not characterize plant macrofossil remains in the soil cores to assess past vegetation successional stages (e.g., Mathijssen et al., 2019). This information could have given us an insightful view of how much the community compositions have changed over the last decades, which is essential to assess how much of the current plant composition relates to actual C pools.

Finally, we obtained high model fits in the undisturbed peatland, showing that these vegetation attributes are reliable proxies of soil C decomposition. This means that vegetation can be used for monitoring C degradation using in‐situ measurements alone or in combination with remote sensing data (Cabezas et al., 2015; Castillo‐Riffart et al., 2017; Harris et al., 2015; Jaenicke et al., 2011; e.g., Lopatin et al., 2019). The latter could provide the means to periodically assess the ecological status of peatlands and its relation to the C cycle at large scales (Ciais et al., 2014; Cole et al., 2013; O’Rourke et al., 2015). Nevertheless, as the human impact on natural ecosystems still increases, it is imperative to find solutions to accurately monitor the C cycle components in semi‐natural and degraded ecosystems.

## CONFLICT OF INTEREST

The authors declare that there are no conflicts of interest.

## AUTHOR CONTRIBUTIONS

**Javier Lopatin:** Conceptualization (equal); Data curation (lead); Formal analysis (lead); Investigation (lead); Methodology (lead); Validation (lead); Visualization (lead); Writing – original draft (lead); Writing – review & editing (lead). **Rocío Araya‐López:** Conceptualization (equal); Writing – original draft (supporting); Writing – review & editing (supporting). **Mauricio Galleguillos:** Funding acquisition (lead); Methodology (supporting); Resources (lead); Writing – original draft (supporting); Writing – review & editing (supporting). **Jorge F. Perez‐Quezada:** Data curation (lead); Funding acquisition (lead); Methodology (supporting); Resources (lead); Writing – original draft (supporting); Writing – review & editing (supporting).

### OPEN RESEARCH BADGES

This article has been awarded Open Materials, Open Data Badges. All materials and data are publicly accessible via the Open Science Framework at https://github.com/JavierLopatin/Peatland‐Disturbance‐C‐Pools.

## Supporting information

Fig S1

Fig S2

Supplementary Material

## Data Availability

The data and R scripts used in this investigation are available from the GitHub repository https://github.com/JavierLopatin/Peatland‐Disturbance‐C‐Pools.
