# Supplementary figures and images for: Disturbance alters relationships between soil carbon pools and aboveground vegetation attributes in an anthropogenic peatland in Patagonia
Source: Ecol Evol. 2022 Mar 21;12(3):e8694. doi: 10.1002/ece3.8694 (PMC8935636; doi:10.1002/ece3.8694)

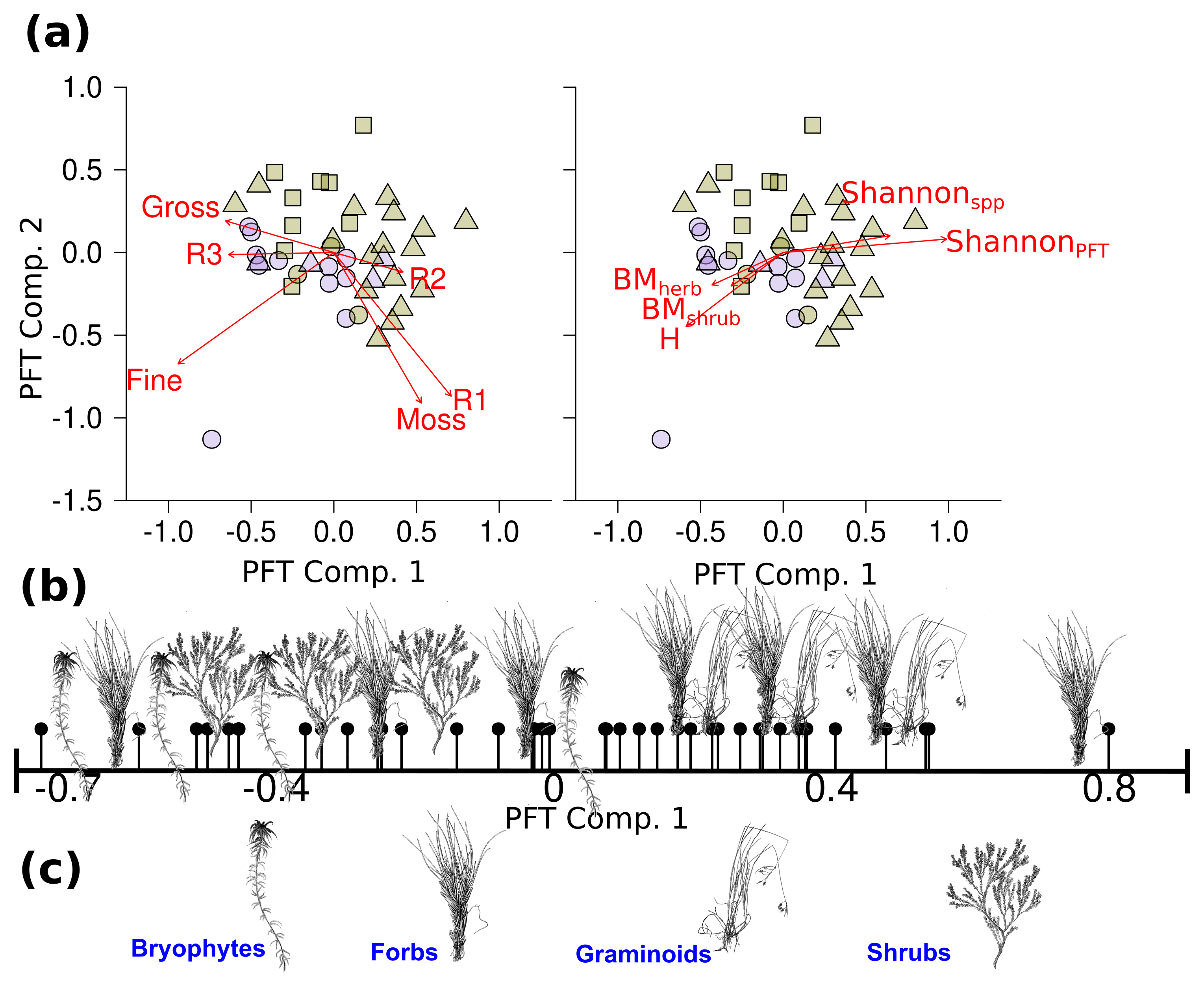

Supplement: Supplementary file 1 — Fig S1 [file ECE3-12-e8694-s002.png]

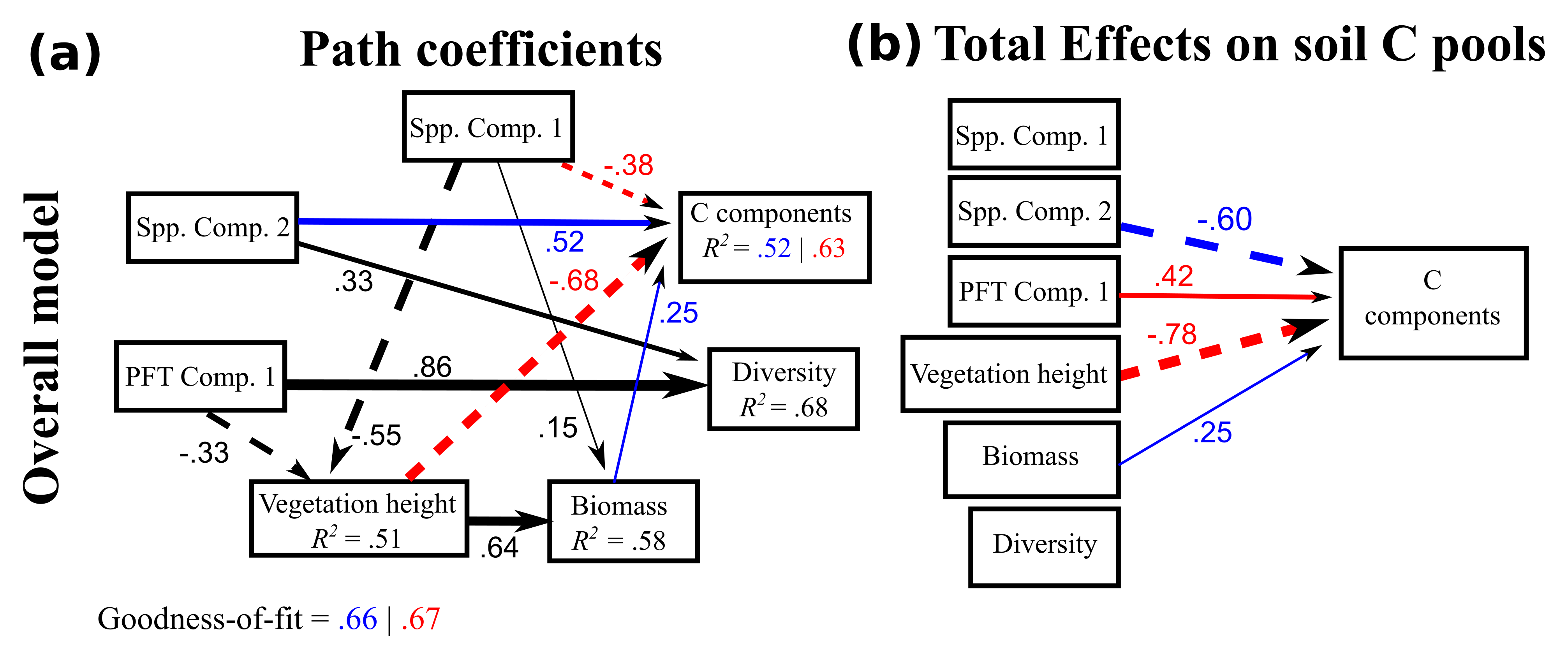

Supplement: Supplementary file 2 — Fig S2 [file ECE3-12-e8694-s001.png]
