# Supplementary material for: Disturbance alters relationships between soil carbon pools and aboveground vegetation attributes in an anthropogenic peatland in Patagonia
Source: Ecol Evol. 2022 Mar 21;12(3):e8694. doi: 10.1002/ece3.8694 (PMC8935636; doi:10.1002/ece3.8694)
Supplement: Supplementary file 3 — Supplementary Material [file ECE3-12-e8694-s003.docx]

APPENDIX S1

Table S1. Summary of the aboveground vegetation attributes.

| Variables | Indicators (unit) | Undisturbed (min−max ± std) | Disturbed (min−max ± std) |
| --- | --- | --- | --- |
| Vascular vegetation height | Vegetation height (cm) | 14.78−130.56 ± 37.30 | 2.83−41.22 ± 9.56 |
| Plant diversity | Species-based Shannon-Wiener index (unitless) | 0.49−1.7 ± 0.35 | 0.59−2.21 ± 0.36 |
|  | PFT-based Shannon-Wiener index (unitless) | 0−1.00 ± 0.28 | 0.2−-1.36 ± 0.30 |
| Species Composition | Spp. Comp. 1 (unitless) | -0.78−-0.17 ± 0.17 | -0.42−1.19 ± 0.44 |
|  | Spp. Comp. 2 (unitless) | -0.69−0.64 ± 0.41 | -1.12−0.71 ± 0.50 |
|  | Spp. Comp. 3 (unitless) | -0.39−0.67 ± 0.24 | -0.66−1.12 ± 0.42 |
| PFT Composition | PFT Comp. 1 (unitless) | -0.74−0.30 ± 0.32 | -0.59−0.80 ± 0.34 |
|  | PFT Comp. 2 (unitless) | -1.12−0.16 ± 0.30 | -0.52−0.78 ± 0.31 |
| Aboveground Biomass | Herbaceous biomass (kg m^-2^) | 0.21−0.86 ± 0.54 | 0.03−0.86 ± 0.21 |
|  | Shrub biomass (kg m^-2^) | 0.01−5.36 ± 1.40 | 0−0.74 ± 0.17 |

Table S2. Results of the multiple regression analysis predicting the first and second C pool components using the species- and PFT-based floristic gradients.

|  | C Comp. 1 | | | | | C Comp. 2 | | | | |
| --- | --- | --- | --- | --- | --- | --- | --- | --- | --- | --- |
|  | Species-based analysis | | | PFT-based analysis | | Species-based analysis | | | PFT-based analysis | |
| NMDS component | 1 | **2** | 3 | **1** | 2 | **1** | 2 | 3 | **1** | 2 |
| Coeff. | 0.37 | **1.06** | -1.15 | **0.81** | -0.39 | **0.51** | -0.15 | 0.18 | **0.36** | -0.303 |
| *t*-value | 1.98 | **5.01** | -0.60 | **2.27** | -1.23 | **3.37** | -1.25 | 1.70 | **2.09** | -1.58 |
| *P*-value | 0.05. | **<0.001***** | 0.55 | **0.029*** | 0.22 | **0.001**** | 0.22 | 0.09. | **0.04*** | 0.12 |

Signif. codes: *** = 0.001; ** = 0.01; * = 0.05; . = 0.1


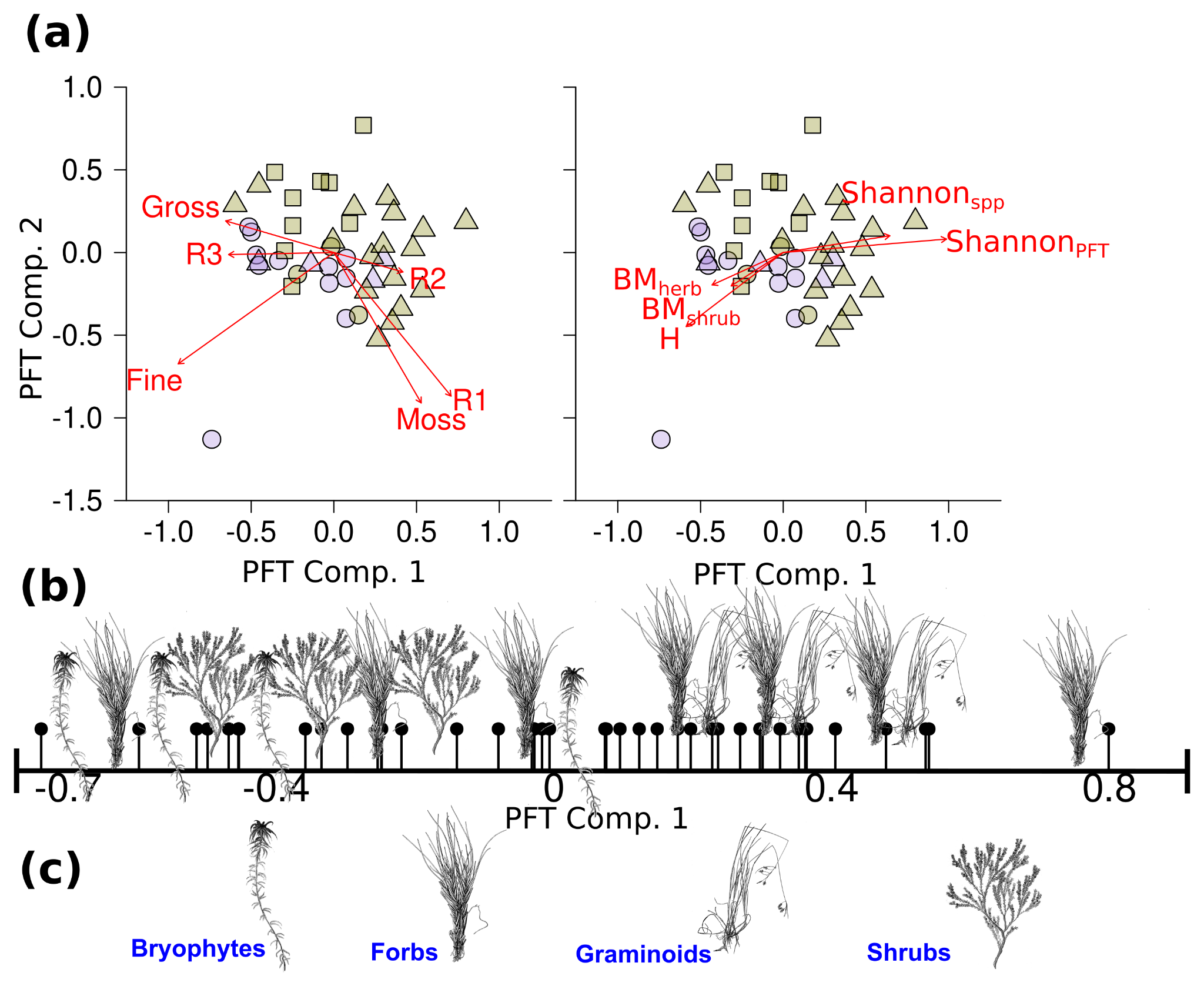


Fig. S1. Peatland PFT-based gradients. (a) Distribution of plots (sample points) in the two-dimensional ordination space of NMDS, showing the delivery of the belowground C stocks (left) and the management types and Isopam classes (right). Close plots feature more similar species composition than remote plots. (b) Location of the most typical species and field-plots (black dots) along the first two NMDS axes. (c) Dominant PFTs were identified with the Isopam clustering algorithms.


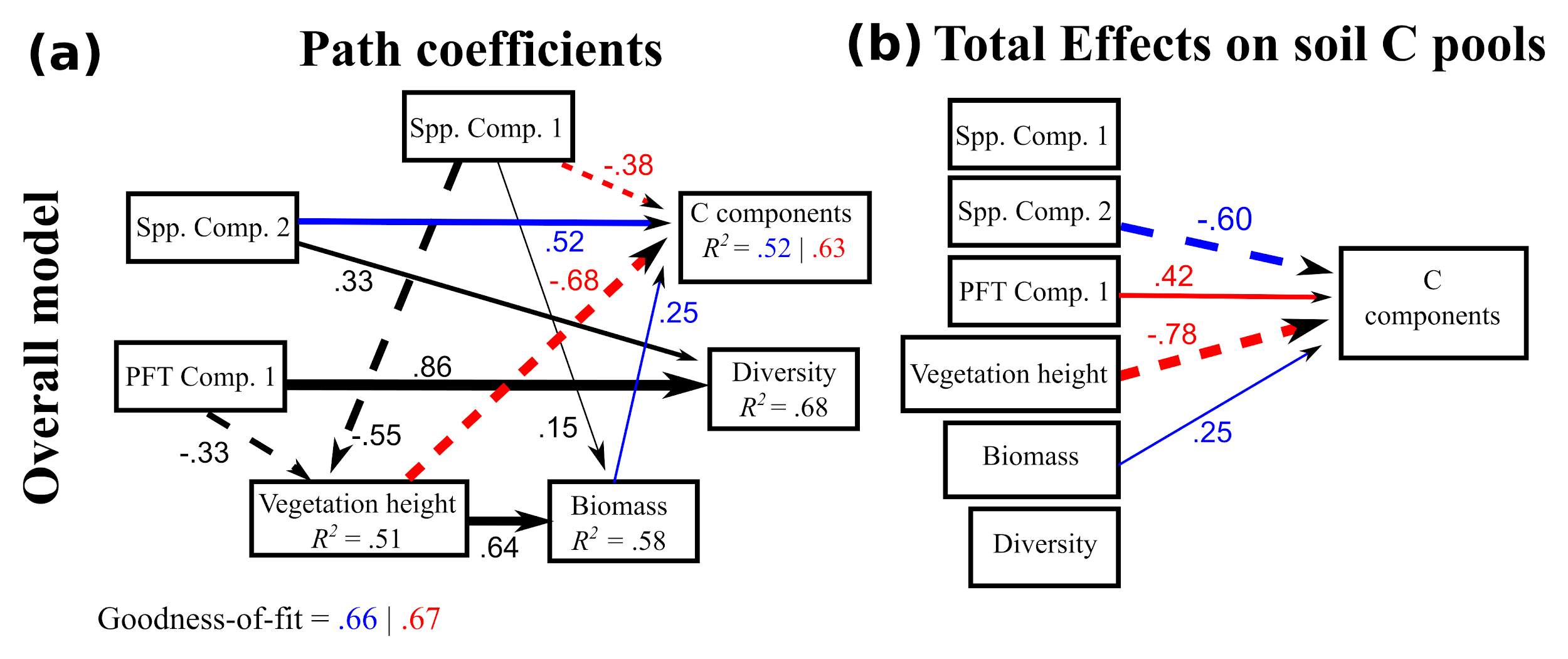


Fig. S2. PLS-PM results for the overall model. Arrows represent significant path coefficients among LVs, where solid and dashed arrows denote positive and negative relationships. Direct effects over the soil C pools are presented in blue and red for the first and second NMDS C components, respectively. Path coefficient thickness is scaled based on its magnitude. Only significant relationships are presented (𝛼 = 0.05).

Table S3. Morans’ *I* test of spatial autocorrelation in the PLS-PM inner model residuals. Grey areas depict predictions with spatial autocorrelation problems (*P*-value < 0.05).

| Latent Variable | Overall model | | Undisturbed model | | Disturbed model | |
| --- | --- | --- | --- | --- | --- | --- |
|  | *I* | *P*-value | *I* | *P*-value | *I* | *P*-value |
| Vegetation Height | -0.01 | 0.46 | -0.08 | 0.71 | -0.06 | 0.33 |
| Aboveground biomass | -0.02 | 0.87 | -0.03 | 0.33 | -0.04 | 0.89 |
| Species diversity | -0.03 | 0.74 | -0.11 | 0.40 | -0.04 | 0.86 |
| C Comp. 1 | -0.04 | 0.30 | -0.06 | 0.92 | -0.09 | 0.04 |
| C Comp. 2 | -0.04 | 0.44 | -0.05 | 0.76 | -0.05 | 0.57 |
